# Glimpse of ATOM in non-human species?

**DOI:** 10.3389/fpsyg.2013.00460

**Published:** 2013-07-23

**Authors:** Christian Agrillo, Maria Elena Miletto Petrazzini

**Affiliations:** Department of General Psychology, University of PadovaPadova, Italy

During the last decade, both behavioral and neuroimaging studies suggested the idea of a common magnitude system for non-symbolic estimation of time, space, and number, the so-called “A Theory Of Magnitude” (ATOM, Walsh, 2003). To date, different approaches have been adopted in cognitive psychology to test the predictions of ATOM. The most common approach is based on behavioral data and uses conflict paradigms (e.g., stroop tasks) to explore the interactions among the three domains (Dormal et al., 2006; Vicario et al., 2008; Agrillo et al., 2010). For instance, in a task requiring to remember different arrays of lines associated to congruent (e.g., small line-short tone) or incongruent (e.g., small line-long tone) temporal information, participants proved able to remember better the spatial–temporal pairings when variation in space and time was congruent (Srinivasan and Carey, 2010). Bisection tasks are also used to study the interaction among magnitudes. It has been shown that our accuracy in a temporal bisection task is significantly affected by changing the numerical size presented on the screen, suggesting that the size of a number influences the subjective midpoint of a duration (Vicario, 2011). Alternatively, reproduction tasks are presented in which participants are told to reproduce the estimated duration of an event (Lu et al., 2009; Chang et al., 2011), the number of items or the length of a line, with the assumption that non-relevant information (e.g., numbers presented on the screen in a spatial task) should not affect the estimation of the relevant information if different systems are involved. Using this paradigm it has been shown that the reproduction of a spatial extension is underestimated when delimited by two small numbers, while it is overestimated if delimited by two large numbers (de Hevia et al., 2008). Neuro-anatomical correlates are also observed to explore the similarity of the three domains. Indeed if the three dimensions are processed by the same cognitive mechanism, similar neural networks should be involved (Cohen Kadosh et al., 2008; Vicario and Martino, 2010). Based on this evidence, several authors have referred to the possibility that we would represent numerical magnitudes using a spatial layout, the so-called “mental number line,” as well as we would represent time flow using a “mental time line” (Daar and Pratt, 2008; Bonato et al., 2012), which again aligns with the idea of a common system for magnitude processing.

Comparative studies showed that temporal, spatial, and numerical abilities are not only a human prerogative. Non-human species have been found to use temporal, spatial and numerical information in both laboratory and field studies (Breukelaar and Dalrymple-Alford, 1999; Evans et al., 2009; Spence et al., 2011). Nonetheless, the debate surrounding ATOM has been almost entirely confined to our species, and it is currently unknown whether the supposed common magnitude system is a recent evolutionary development (restricted to humans) or, in contrast, is more evolutionarily pervasive. However, even in the absence of studies directly focused on testing the predictions of ATOM, we believe that the hallmark of ATOM can be tracked in comparative literature.

First of all, time, space, and number estimation obeys Weber's law in most species. The capacity to discriminate between two quantities (durations, areas or number of items) becomes increasingly accurate as the ratio between the smaller and the larger quantity decreases (time: Gibbon, 1977; space: Cheng, 1990; number: Perdue et al., 2012). The universality of Weber's law reinforces the idea of similar quantificational systems among species (Beran, 2008) and highlights the similarities in cognitive processes for time, space, and number estimation, which aligns with the idea of a common magnitude system.

More compelling evidence comes from studies directly presenting two different magnitudes and using contrast paradigms similar to those adopted in human literature. For instance, both mammals (rats) and birds (pigeons) show number-time interaction. Meck and Church (1983) initially trained rats to discriminate auditory stimuli varying in both number and duration (i.e., 2 sounds in 2 s or 8 sounds in 8 s). Subsequently the subjects were tested in a transfer task, where novel stimuli varying in just one dimension were presented (2–8 tones in 4 s or 4 tones in 2–8 s). The rats spontaneously encoded information about both time and number, thus showing an almost identical psychophysical function for both time and number (Meck and Church, 1983). Roberts and Mitchell (1994) obtained similar results with pigeons trained to discriminate between sequences of light flashes differing in number and duration. Pigeons, like rats, were able to process both number and time simultaneously. Apparently, both species cannot ignore the non-relevant information (i.e., temporal information in a numerical task) in both temporal and numerical tasks. Glimpse of ATOM can be found also in research on time-space interactions. In two different experiments, Merritt et al. (2010) tested rhesus monkeys in contrast paradigms. In one of these tasks (duration bisection), the subjects had to judge seven durations (two anchors and five intermediate values) as long or short while the length of different lines was orthogonally varied between short, medium, and long values. The authors found that irrelevant spatial information affected time judgments, and irrelevant temporal information affected space judgments, in agreement with ATOM. A recent study also found that non-human primates use a common or partially overlapping neural network to represent spatial and temporal information (Mendez et al., 2011).

Most of the literature has focused on number-space interactions, but, here, the picture is less clear. Part of the problem is due to the fact that comparative psychologists working in numerical or spatial cognition have seldom discussed their data through the prism of ATOM. For instance, when studying numerical abilities in a non-human species, researchers initially present stimuli differing in numerosity (i.e., 2 vs. 3 food items) without control of non-numerical continuous quantities that co-vary with number, such as cumulative surface area or the overall space occupied by the arrays. If subjects accomplish the task, only then a subsequent test is set up to control for continuous quantities in order to see whether the subjects display the ability to use number only. Even though these studies are commonly done with the main purpose to control for non-numerical cues, we believe that their results may also have interesting implications for the theoretical debate surrounding ATOM. For instance, in a 6 vs. 12 dot discrimination, numerical and spatial cues are initially congruent (1:2 numerical ratio and 1:2 area ratio) whereas in the following “true” numerical test, there is not a congruence between numerical and spatial cues (1:2 numerical ratio and 1:1 area ratio). Any performance decrease in the “number only” condition could provide important clues for ATOM; indeed this performance decrease can be used to infer the existence of a single system, exactly as cognitive psychologists use contrast paradigms in human studies.

For instance, Pisa and Agrillo (2009) trained cats to discriminate between two groups of dots differing in numerosity (2 vs. 3) in order to get a food reward. During the initial training, stimuli were not controlled for continuous quantities and both numbers and continuous quantities could be used to select the larger/smaller group. After the cats have reached the learning criterion, stimuli controlled for continuous spatial cues were presented: the cats' performance dropped to chance level. Similarly, Krusche et al. (2010) tested whether salamanders can discriminate between two groups of 8 and 16 crickets when number and continuous (spatial) cues were simultaneously available. The amphibians were able to discriminate between the two quantities; however, when stimuli were controlled for spatial cues, their performance decreased. More recently Agrillo et al. (2011) analyzed the learning rates of trained mosquitofish for numerical and spatial discrimination. Fish were required to discriminate between two quantities of figures in order to select the correct tunnel and re-join their conspecifics. In details, they were required to discriminate 2 items from 3 in three different tests. In test 1, continuous quantities were controlled while numerical information was available (they could use only numerical information); in test 2, the number was kept constant (1 vs. 1) and information relating to continuous variables was available (only continuous quantities could be used); in the third test, stimuli differed for both numbers and continuous quantities (they could use both information). Fish learned to discriminate more quickly when both numbers and continuous quantities were available compared to when they could use continuous quantities only or numbers only; interestingly, no difference in the learning rate between the two latter conditions was found. Hence, when number and space are experimentally contrasted (test 1 and 2) fish performance was less accurate.

Nonetheless other comparative studies have not found any performance decrease when numerical and spatial cues were contrasted, making the picture more complex. Flombaum et al. (2005) showed that rhesus monkeys can discriminate between 4 and 8 lemons both when numerical and continuous (spatial) information were simultaneously available, and when continuous quantities were controlled for, with apparently the same accuracy in both conditions. Cantlon and Brannon (2007) trained rhesus monkeys to discriminate between groups of bi-dimensional figures differing in numerosity. Stimuli were not controlled for spatial cues, therefore numerical and spatial cues were both available during the learning phase. In the test phase, number was pitted against a spatial cue (cumulative surface area): a monkey without any experience in the numerical task was more affected by cumulative surface area whereas number-experienced monkeys primarily used numerical information without showing any decrease in performance. These findings contradict a strong version of ATOM. Trained pigeons also do not show any difference in accuracy when numerical and spatial information are simultaneously available and when only number can be used (Emmerton and Renner, 2006).

We are aware that none of these studies on numerical abilities were directly planned to test the predictions of ATOM; therefore, conclusions can be made only with caution. However, we believe that data on temporal, spatial and numerical cognition hint at the existence of a shared magnitude system in non-human species, either for all three or for a sub-set of magnitudes. To date, most of the inconsistencies have been reported in the research field of number-space interaction. We prefer not to speculate on this latter point as this may simply reflect the fact that number-space interactions have been investigated more during the last decade than number-time or time-space interactions; as a consequence, more data are available in this research field with inter-species variations and a wide range of experimental procedures.

Further investigation is needed to shed light on the evolutionary roots of ATOM. Is ATOM shared among vertebrates or are there inter-species differences in processing time, space and number? Different selective pressures might have shaped the neuro-cognitive systems underlying magnitude estimation. If so, it will be challenging to investigate which selective constraints have shaped ATOM. The possibility also remains that a weaker version of ATOM might be more appropriate in non-human species. Separate stimulus-processing pathways (for instance, one for time-number and one for time-space) might exist in non-human animals as advanced in our species (Agrillo et al., 2010). Also, not all aspects of time, space, and numbers are supposed to have a common origin, as advocated by Walsh himself (2003). Non-human species may display multiple core systems for each domain (Miletto Petrazzini et al., 2013) and only a sub-set of them may be processed by a shared system for the three magnitudes. For instance, Breukelaar and Dalrymple-Alford (1999) found that lesions to the cerebellar hemispheres of rats produced performance deficits in a numerical discrimination task (2–8 events) and a ms temporal discrimination task (0.2–0.8 s). In contrast, temporal discriminations in the seconds range (2–8 s) were unaffected by the lesion, suggesting the existence of two independent cognitive systems for time processing. Distinct mechanisms for subsecond and suprasecond timing in rats have been also reported by Cordes and Meck (2013). Similarly, separate numerical systems for small (<4) and large numbers have been hypothesized in human and non-human species (Cutini and Bonato, 2012; Piffer et al., 2012).

To date, there is no study that has investigated more than two interactions in the same species (i.e., number-space, time-number, time-space), a study that may provide us with useful insights. Reproduction (e.g., monkeys requiring to push a button as long as they estimate the duration of an event) and bisection tasks (e.g., selecting the midpoint of a given interval) are also required, as the exclusive use of stroop-like paradigms as a tool to investigate the interaction between two magnitudes has been criticized (see Vicario, 2012, 2013). Nobody has studied individual differences in time, space and number estimation. One potential prediction from ATOM is that increased abilities in one domain should determinate increased abilities in another. A very recent study showed that musicians outperformed non-musicians in temporal, spatial and numerical tasks (Agrillo and Piffer, 2012): it was hypothesized that the increased temporal abilities due to long-term musical training led them to improve their performance even in tasks involving different magnitudes (space and number). The study of individual differences and expertise (Agrillo, 2013) in the trinity of magnitude advanced by Walsh (2003) might help us to form a broader comprehension of the cognitive systems supporting time, space and number in non-human species.
